# Music processing similarities between sleeping newborns and alert adults: cause for celebration or concern?

**DOI:** 10.3389/fpsyg.2013.00644

**Published:** 2013-09-19

**Authors:** Sandra E. Trehub

**Affiliations:** Department of Psychology, University of Toronto Mississauga Mississauga, ON, Canada

**Keywords:** music perception, newborns, interpretations, adult comparisons, infants

The proliferation of newborn “musical” abilities is accelerating with the ever-increasing use of neurophysiological methods with sleeping newborns. Newborn abilities that have surfaced in recent years include beat detection (Winkler et al., 2009), representation of pitch independent of timbre (Háden et al., 2009), representation of pitch intervals independent of absolute pitch (Stefanics et al., 2009), detection of changes in tonal key (Perani et al., 2010), and lateralized responses to speech and music (Kotilahti et al., 2010; Perani et al., 2010). Virtala et al. (2013) add to this body of work with their report of newborns' sensitivity to major vs. minor chords and consonant vs. dissonant chords.

Unquestionably, the newborn brain registers the aforementioned differences in some manner, but does such registration enrich our knowledge of music processing and its development? The answer is affirmative but with important reservations. Newborns in Virtala et al. (2013) responded to chords on the basis of relative rather than absolute pitch cues, confirming previous neurophysiological and behavioral evidence of relational processing in newborns (Stefanics et al., 2009) and older infants (e.g., Plantinga and Trainor, 2005).

What about the reservations? The authors' interpretation of newborns' neural responses to the major/minor distinction raises serious concerns. They argue that newborn responses were based on major or minor *chord quality* rather than mere acoustic differences. Infants could have responded to simple differences in interval size, as the authors acknowledge, because the lower interval of the minor chord (minor third, 3 semitones) is smaller than that of the major chord (major third, 4 semitones). If newborns really differentiate major from minor chord quality, as the authors contend, then why do preschoolers (Costa-Giomi, 1996) and musically untrained 13-year-olds (Virtala et al., 2012) fail to do so? The authors suggest that early sensitivity to the major/minor distinction could disappear in the absence of musical training, but it is unclear what would prompt such a disappearance. Loss of sensitivity to specific phonetic distinctions has been reported for distinctions that are irrelevant to the language being learned (Werker and Tees, 2005). The major/minor distinction, although not universal, is surely relevant to Western music and to the young children who fail to notice it.

The authors' interpretation of newborns' responsiveness to the consonance/dissonance distinction poses further problems. They suggest that sensitivity to this distinction can be regarded as a biological predisposition for musical skills. As with the major/minor distinction, newborns may have responded on the basis of the smaller lower interval (minor seconds or 2 semitones) in the dissonant chords than in the consonant chords (major thirds or 4 semitones). Sensitivity to the consonance/dissonance distinction has been reported in other species (e.g., Hulse et al., 1995), but no one is suggesting that birds have biological predispositions for music. In this instance, at least, what's good for the birds should be good for babies.

The present investigators are not alone in their generous interpretation of the infant findings. For example, newborns' apparent sensitivity to musical key (Perani et al., 2010) is out of line with children's insensitivity to key structure until 4 or 5 years of age (Corrigall and Trainor, 2010). Moreover, newborns' reported ability to process pitch independent of timbre (Stefanics et al., 2009) is at odds with adults' difficulty comparing pitches in the context of contrasting timbre (Borchert et al., 2011). By contrast, reports of beat detection in newborns (Winkler et al., 2009) are consistent with skills that infants display some months later (with different methods), for example, rhythm perception influenced by rhythmic movement (Phillips-Silver and Trainor, 2005) and preferences for native over non-native musical meters (Soley and Hannon, 2010).

It is understandable that parents often provide very rich interpretations of their infants' and young children's abilities. There is less justification for comparably rich interpretations from scholars who study music perception in sleeping newborns. As scholars, we must remain on guard against anthropomorphism and ethnocentrism. We must be equally vigilant about adultomorphism.
